# Supplementary material for: Label-free morpho-molecular phenotyping of living cancer cells by combined Raman spectroscopy and phase tomography
Source: Commun Biol. 2024 Jun 29;7:785. doi: 10.1038/s42003-024-06496-9 (PMC11217291; doi:10.1038/s42003-024-06496-9)
Supplement: Supplementary file 2 — Supplementary Information [file 42003_2024_6496_MOESM2_ESM.pdf]

Supplementary Information

Label-free Morpho-Molecular Phenotyping of Living Cancer Cells by Combined Raman Spectroscopy and Phase Tomography

Arianna Bresci[1,2 *], Koseki J. Kobayashi-Kirschvink[1,3], Giulio Cerullo[2,4], Renzo Vanna[4], Peter T. C. So[1,5,6], Dario Polli[2,4 *], Jeon Woong Kang[1 *]

[1] *G. R. Harrison Spectroscopy Laboratory, Massachusetts Institute of Technology, Cambridge, Massachusetts, 02139, USA*

[2] *Department of Physics, Politecnico di Milano, Milan, 20133, Italy*

[3] *Klarman Cell Observatory, Broad Institute of MIT and Harvard, Cambridge, Massachusetts, 02142, USA*

[4] *CNR-Institute for Photonics and Nanotechnologies (CNR-IFN), Milan, 20133, Italy.*

[5] *Department of Mechanical Engineering, Massachusetts Institute of Technology, Cambridge, Massachusetts, 02139, USA*

[6] *Department of Biological Engineering, Massachusetts Institute of Technology, Cambridge, Massachusetts, 02139, USA*

* Correspondence: abresci@mit.edu (A.B.), dario.polli@polimi.it (D.P.), jwkang76@mit.edu (J. W. K.)

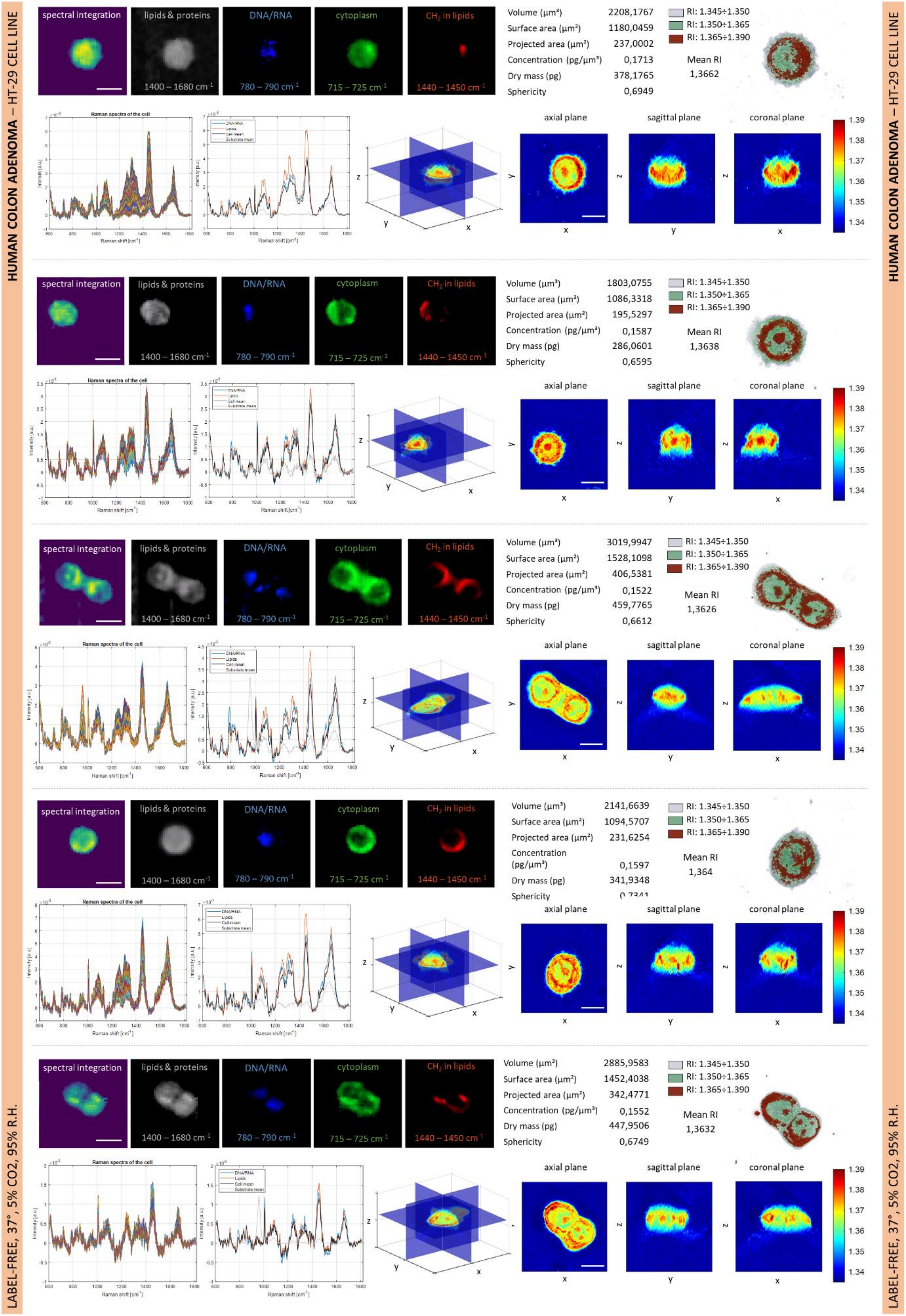

HUMAN COLON ADENOMA – HT-29 CELL LINE
LABEL-FREE, 37°, 5% CO2, 95% R.H.
spectral integration
lipids & proteins
1400 – 1680 cm-1
DNA/RNA
780 – 790 cm-1
cytoplasm
715 – 725 cm-1
CH2 in lipids
1440 – 1450 cm-1
Raman spectra of the cell
Intensity [a.u.]
Raman shift [cm-1]
DNA/RNA
Lipids
Cell mean
Substrate mean
axial plane
sagittal plane
coronal plane
RI: 1.345÷1.350
RI: 1.350÷1.365
RI: 1.365÷1.390
Volume (µm³) 2208,1767
Surface area (µm²) 1180,0459
Projected area (µm²) 237,0002
Concentration (pg/µm³) 0,1713
Dry mass (pg) 378,1765
Sphericity 0,6949
Mean RI 1,3662
Volume (µm³) 1803,0755
Surface area (µm²) 1086,3318
Projected area (µm²) 195,5297
Concentration (pg/µm³) 0,1587
Dry mass (pg) 286,0601
Sphericity 0,6595
Mean RI 1,3638
Volume (µm³) 3019,9947
Surface area (µm²) 1528,1098
Projected area (µm²) 406,5381
Concentration (pg/µm³) 0,1522
Dry mass (pg) 459,7765
Sphericity 0,6612
Mean RI 1,3626
Volume (µm³) 2141,6639
Surface area (µm²) 1094,5707
Projected area (µm²) 231,6254
Concentration (pg/µm³) 0,1597
Dry mass (pg) 341,9348
Sphericity
Mean RI 1,364
Volume (µm³) 2885,9583
Surface area (µm²) 1452,4038
Projected area (µm²) 342,4771
Concentration (pg/µm³) 0,1552
Dry mass (pg) 447,9506
Sphericity 0,6749
Mean RI 1,3632
1.39
1.38
1.37
1.36
1.35
1.34

Supplementary Figure 1. Dataset of the morpho-molecular information extracted from single HT29 human colon adenoma cells. RS and TPM imaging were employed on the same FOV in living and label-free cell cultures, and we report here the 5 HT29 cells used in the following regression modeling for cell type inference and discrimination (each single-cell morpho-molecular information is organized in vertically stacked panels). The extracted data consist in Raman spectra in the fingerprint region (Ω = 600 – 1800 cm^{-1}), which can be used to produce false-color chemical images of cells by selecting specific Raman bands (left hand side of each single-cell panel). TPM delivers 3D RI tomogram of cells, which we sliced along the axial, sagittal and coronal plane. By RI thresholding and voxel integration, we obtained relevant quantities describing cell morphology (right hand side of each single-cell panel).

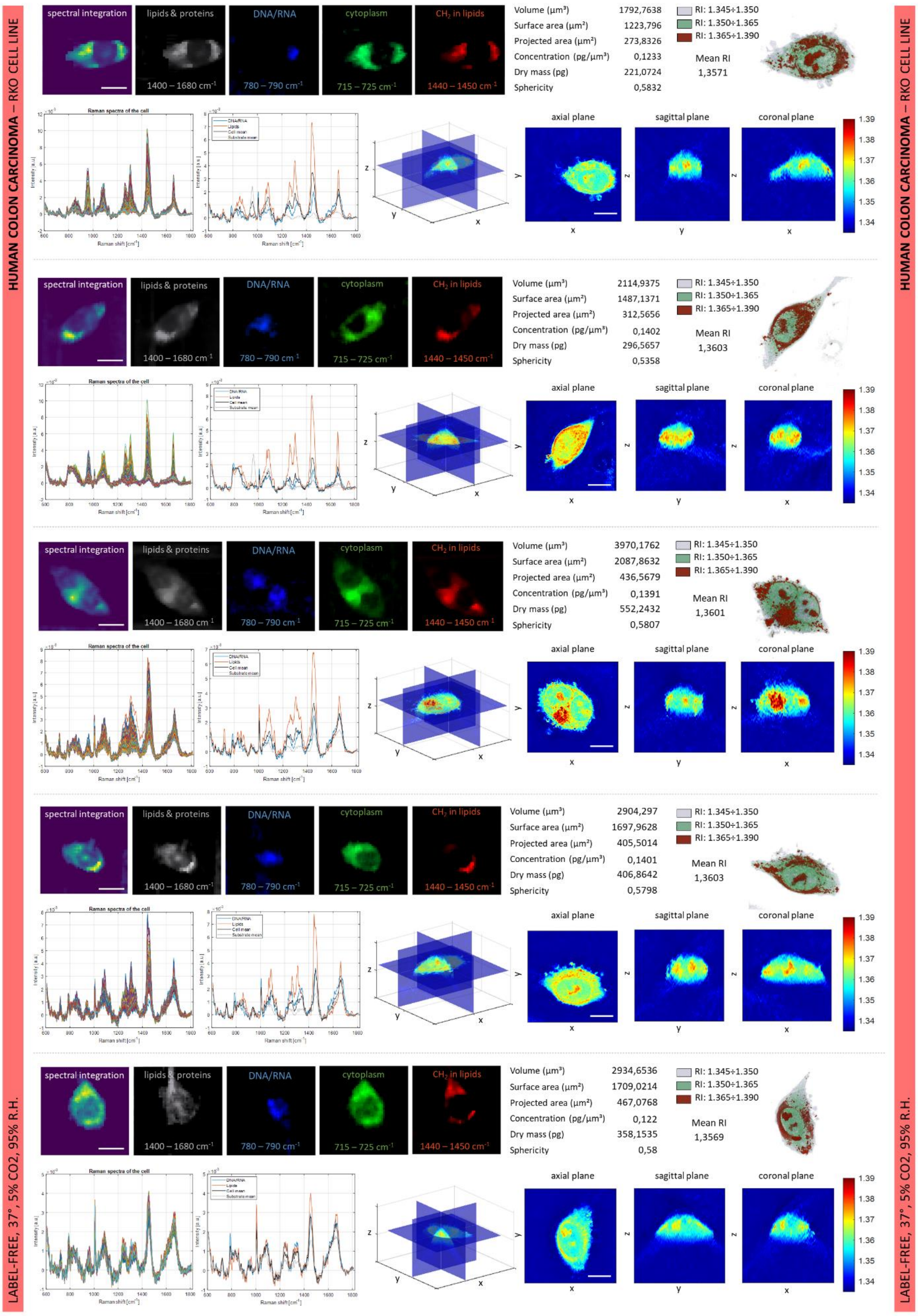

HUMAN COLON CARCINOMA – RKO CELL LINE
LABEL-FREE, 37°, 5% CO2, 95% R.H.
spectral integration
lipids & proteins
1400 – 1680 cm-1
DNA/RNA
780 – 790 cm-1
cytoplasm
715 – 725 cm-1
CH2 in lipids
1440 – 1450 cm-1
Volume (μm³) 1792,7638
Surface area (μm²) 1223,796
Projected area (μm²) 273,8326
Concentration (pg/μm³) 0,1233
Dry mass (pg) 221,0724
Sphericity 0,5832
RI: 1.345÷1.350
RI: 1.350÷1.365
RI: 1.365÷1.390
Mean RI 1,3571
Raman spectra of the cell
Raman shift [cm-1]
axial plane
sagittal plane
coronal plane
1.39
1.38
1.37
1.36
1.35
1.34
Volume (μm³) 2114,9375
Surface area (μm²) 1487,1371
Projected area (μm²) 312,5656
Concentration (pg/μm³) 0,1402
Dry mass (pg) 296,5657
Sphericity 0,5358
Mean RI 1,3603
Volume (μm³) 3970,1762
Surface area (μm²) 2087,8632
Projected area (μm²) 436,5679
Concentration (pg/μm³) 0,1391
Dry mass (pg) 552,2432
Sphericity 0,5807
Mean RI 1,3601
Volume (μm³) 2904,297
Surface area (μm²) 1697,9628
Projected area (μm²) 405,5014
Concentration (pg/μm³) 0,1401
Dry mass (pg) 406,8642
Sphericity 0,5798
Mean RI 1,3603
Volume (μm³) 2934,6536
Surface area (μm²) 1709,0214
Projected area (μm²) 467,0768
Concentration (pg/μm³) 0,122
Dry mass (pg) 358,1535
Sphericity 0,58
Mean RI 1,3569

Supplementary Figure 2. Dataset of the morpho-molecular information extracted from single RKO human colon carcinoma cells. RS and TPM imaging were employed on the same FOV in living and label-free cell cultures, and we report here the 5 RKO cells used in the following regression modeling for cell type inference and discrimination (each single-cell morpho-molecular information is organized in vertically stacked panels). The RS and TPM information here shown is aligned with the one described for HT29 cells, please refer to the description of Supplementary Figure 1 for further detail.

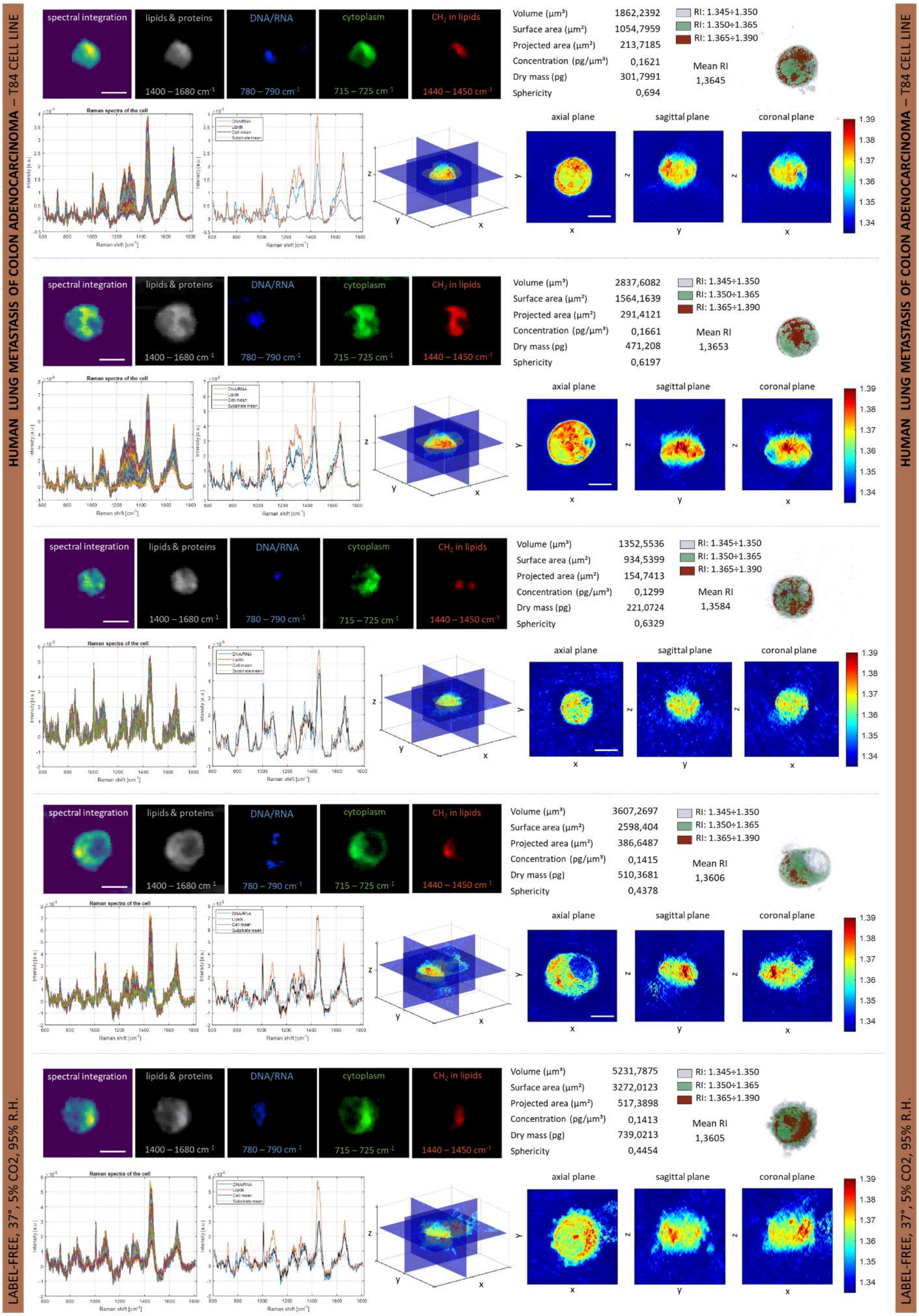
HUMAN LUNG METASTASIS OF COLON ADENOCARCINOMA – T84 CELL LINE
LABEL-FREE, 37°, 5% CO2, 95% R.H.
spectral integration
lipids & proteins
1400 – 1680 cm-1
DNA/RNA
780 – 790 cm-1
cytoplasm
715 – 725 cm-1
CH2 in lipids
1440 – 1450 cm-1
Volume (μm³) 1862,2392
Surface area (μm²) 1054,7959
Projected area (μm²) 213,7185
Concentration (pg/μm³) 0,1621
Dry mass (pg) 301,7991
Sphericity 0,694
RI: 1.345÷1.350
RI: 1.350÷1.365
RI: 1.365÷1.390
Mean RI 1,3645
Raman spectra of the cell
Raman shift [cm-1]
Intensity [a.u.]
DNA/RNA
Lipids
Cell mean
Substrate mean
axial plane
sagittal plane
coronal plane
1.39
1.38
1.37
1.36
1.35
1.34
Volume (μm³) 2837,6082
Surface area (μm²) 1564,1639
Projected area (μm²) 291,4121
Concentration (pg/μm³) 0,1661
Dry mass (pg) 471,208
Sphericity 0,6197
Mean RI 1,3653
Volume (μm³) 1352,5536
Surface area (μm²) 934,5399
Projected area (μm²) 154,7413
Concentration (pg/μm³) 0,1299
Dry mass (pg) 221,0724
Sphericity 0,6329
Mean RI 1,3584
Volume (μm³) 3607,2697
Surface area (μm²) 2598,404
Projected area (μm²) 386,6487
Concentration (pg/μm³) 0,1415
Dry mass (pg) 510,3681
Sphericity 0,4378
Mean RI 1,3606
Volume (μm³) 5231,7875
Surface area (μm²) 3272,0123
Projected area (μm²) 517,3898
Concentration (pg/μm³) 0,1413
Dry mass (pg) 739,0213
Sphericity 0,4454
Mean RI 1,3605

Supplementary Figure 3. Dataset of the morpho-molecular information extracted from single T84 human colon carcinoma cells. RS and TPM imaging were employed on the same FOV in living and label-free cell cultures, and we report here the 5 T84 cells used in the following regression modeling for cell type inference and discrimination (each single-cell morpho-molecular information is organized in vertically stacked panels). The RS and TPM information here shown is aligned with the one described for T84 cells, please refer to the description of Supplementary Figure 1 for further detail.

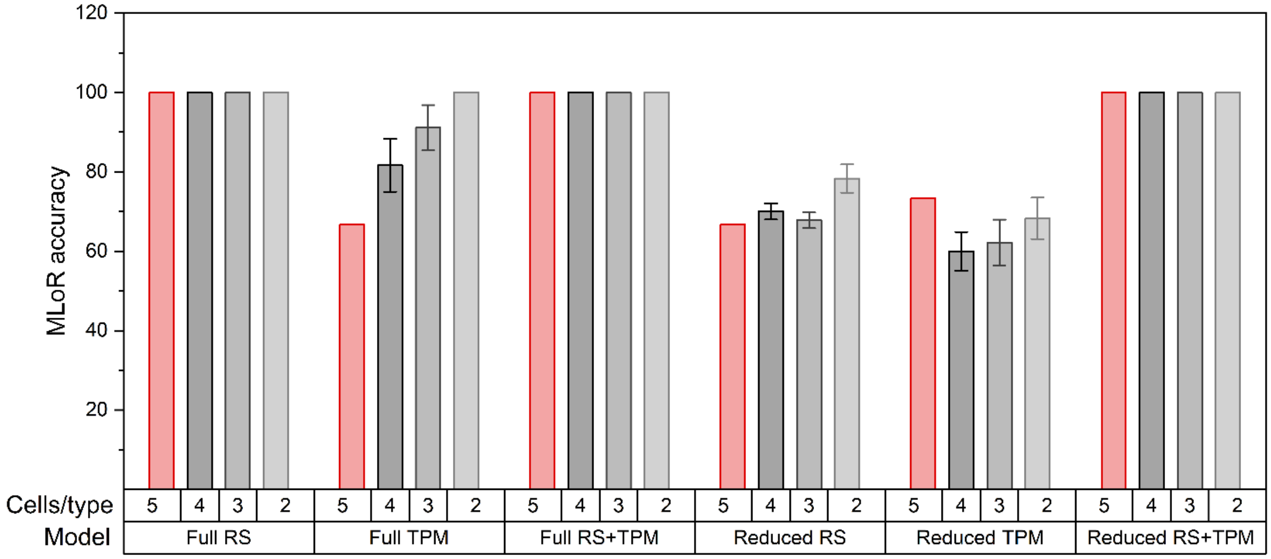

Supplementary Figure 4. MLoR phenotype inference accuracy when varying the number of training cells per type. We report MLoR phenotype inference accuracy when varying the number of cells per type in the training dataset. The full TPM, reduced TPM and reduced RS models show signs of overfitting when using 2 cells per type, witnessed by an appreciable increase in accuracy values with respect to using a higher number of cell examples. By using > 3 cells per each colon cancer type, we did not quantify any significant variation in MLoR phenotype inference. Hence, we chose to use 5 cells per phenotype to speed up the cell characterization process by using a limited number of biological material, while mitigating model overfitting effects that would compromise the soundness of cell type inference. We consider this choice an optimal tradeoff that takes into account cell biological variability while restraining the amount of biological material needed for a quick cell characterization and phenotype analysis. Further confirmatory evidence of the sufficiency of the sample size used through this work can be found in Supplementary Figure 5, which directly studies the intra and inter-phenotype variance of the morpho-molecular variables used to train the full and reduced MLoR inference models.

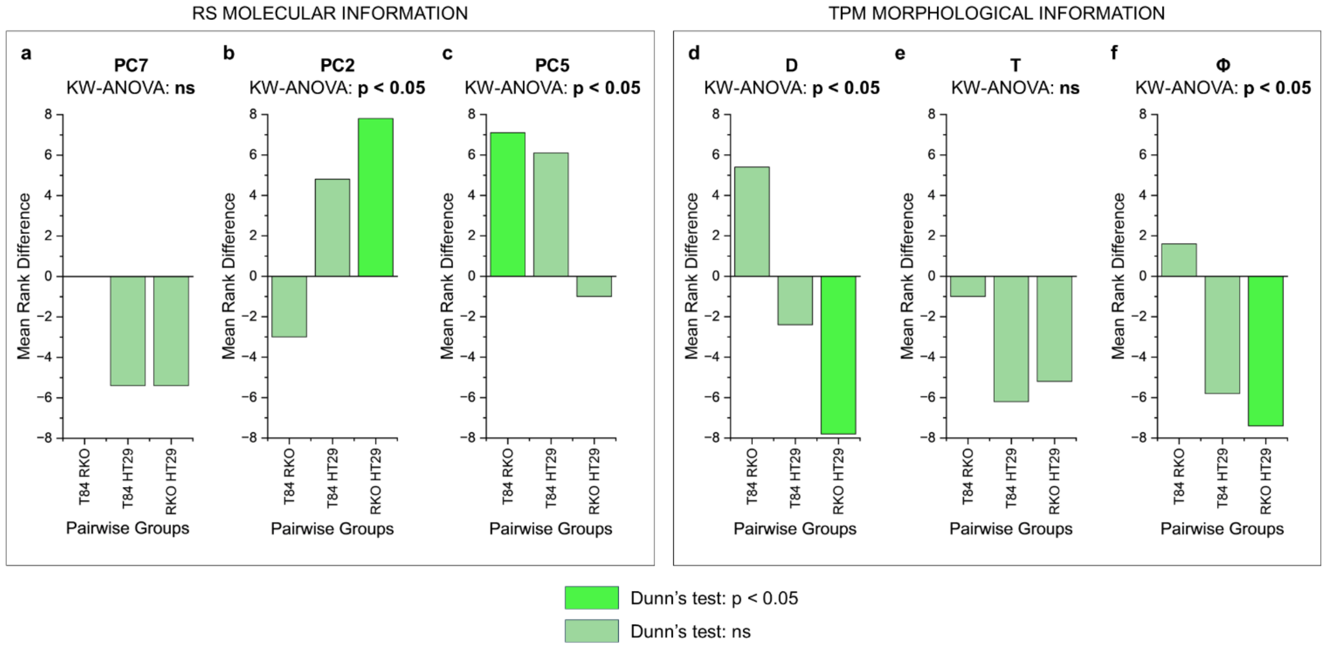

Supplementary Figure 5. The non-parametric Kruskal-Wallis Analysis Of Variance (KW-ANOVA) test of the morpho-molecular observables mostly distinguishing colon cancer phenotypes confirms the sufficiency of a reduced sample size. We applied the KW-ANOVA test to the morpho-molecular observables used to train extended and reduced MLoR inference and prediction models, selected based on their descriptive statistics: **a** PC_2, **b** PC_5 and **c** PC_7 for the RS-based chemical information, and **d** D, **e** Φ, and **f** Z, for the TPM-based morphological information. In the KW-ANOVA test, if the p-value is less than the significance level (0.05), one can reject the null hypothesis and conclude that there are significant differences among at least some of the group means. The KW-ANOVA test exact p-values are: **a** $p = 0.088$, **b** $p = 0.02$, **c** $p = 0.05$, **d** $p = 0.018$, **e** $p = 0.063$, **f** $p = 0.022$. To graphically visualize the statistical differences achieved with our chosen sample size (*i.e.*, five living cancer cells per phenotype), we produced mean rank paired comparisons plots for each morphological or molecular trait analyzed. Thanks to such plots, one can easily visualize which groups are driving the statistical difference in the KW-ANOVA test, if any: the higher the mean rank difference between two phenotypes, the higher the contribution of that cell type pair in driving the overall significance of the KW-ANOVA test. In addition,

we carried out a Dunn's test on the pairwise comparisons: this post-hoc non-parametric test compares all possible pairs of groups using pairwise Mann-Whitney U tests, then corrects the p-values for multiple comparisons via a Bonferroni correction. This test is highly conservative, it strongly limits the false-positive discovery rate of statistical significance and may generate false-negatives. Despite this, and with a limited sample size, our analysis shows that two observables in both the RS-based and TPM-based datasets feature clear statistically significant differences among cell phenotypes. We conclude that the KW-ANOVA and Dunn's tests confirm the sufficiency of our sample size for phenotyping. Due to the high amount of quantitative information that multimodal RS and TPM can extract from each single living cell, these tools are suited for phenotyping tasks even when the biological material is in short supply.

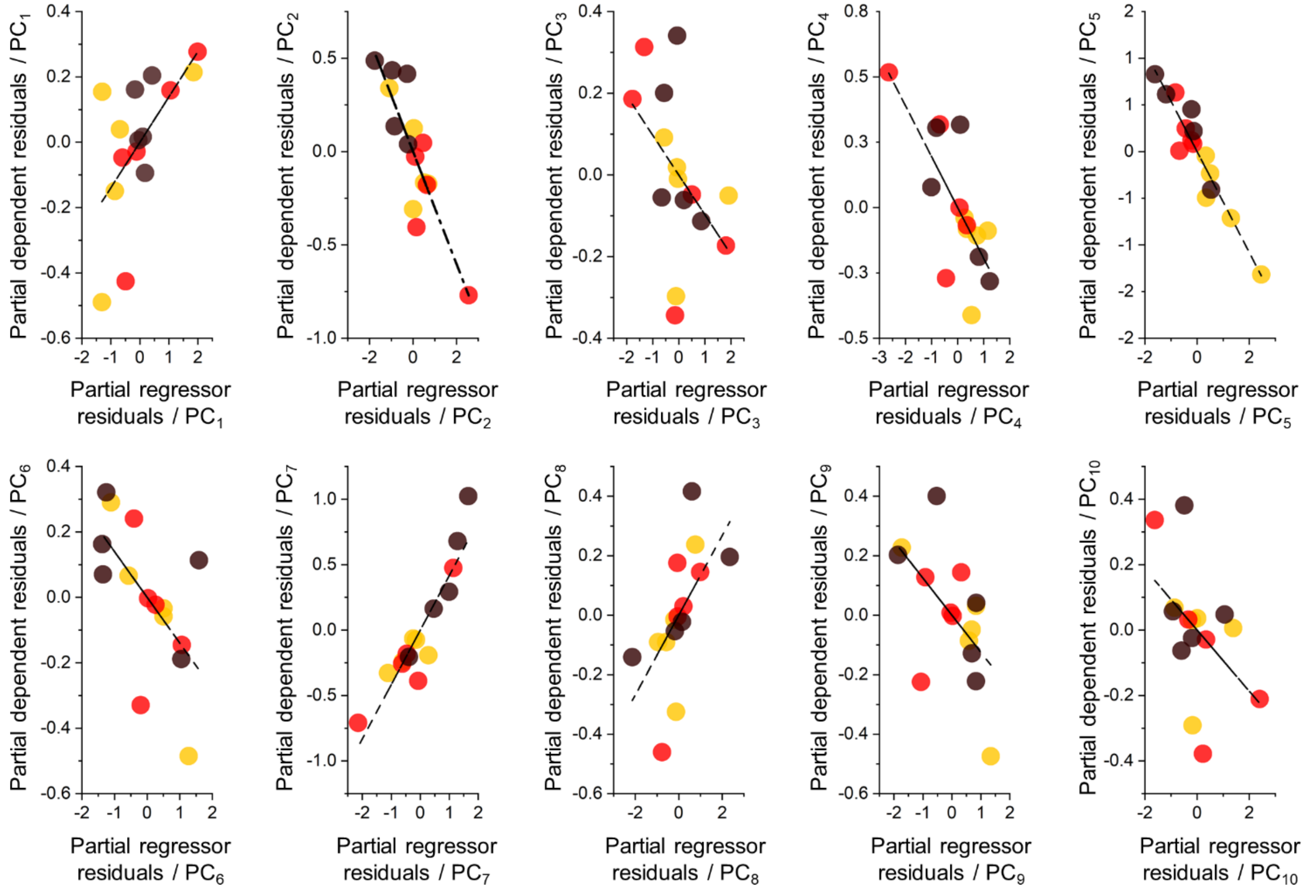

Supplementary Figure 6. PCA-MLR partial leverage plots. The first 10 PCs are used as independent variables to fit an MLR model inferring colon cancer-derived cell phenotypes, namely, HT29 (yellow dots), RKO (red dots), or T84 (brown dots) cell types. For each explanatory independent variable, partial leverage plots are obtained as the residuals of the dependent variable, obtained by omitting the selected regressor, on the y-axis, against the residuals of the selected explanatory variable regressed on all the other independent variables, on the x-axis. The slope of the regression line in partial leverage plots corresponds to the predicted β_i coefficient value in the MLR model (*i.e.*, cell phenotype = $\beta_0 + \beta_1 \cdot PC_1 + \beta_2 \cdot PC_2 + \ldots + \beta_n \cdot PC_n$). Hence, the higher its slope, the higher the significance of β_i in testing against the null hypothesis, leading to the rejection of its null value and the consideration of PC_i as a relevant regressor for the phenotyping task. Figure 3a in the main text reports the value and significance for the

β coefficients of the MLR model: statistical significance is reached by β_2, β_5, and β_7. Notably, only for these regression coefficients, partial leverage plots display data points that are well fit by a line. Residual values distant from the regression line, or, similarly, high-leverage points, which alone impact strongly on the overall slope, are present in all the other regressors, indicating their poor performance in being explanatory variables for phenotype inference.

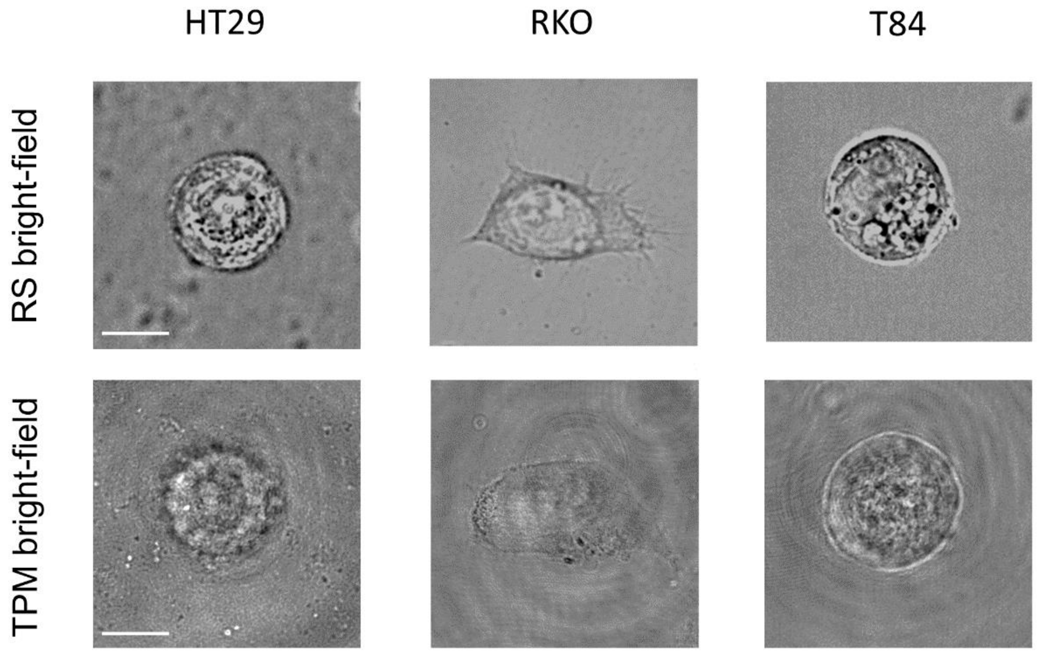

Supplementary Figure 7. Representative bright-field images of human colon cancer-derived cell types. The images are recorded in the RS and TPM systems, sequentially, considering the same FOV. Co-registration of the FOV across microscopy systems is made possible using grid guidance lines on quartz-bottom petri dishes for cell cultures (see Methods, Cell culture). When considering the XY-plane view of cancer cells, it is qualitatively evident that the morphology of RKO types differs from HT29 and T84, being more elongated. Such cells typically feature cytoplasmic extroflexions, which we observed undergo a more evident shape rearrangement when shifting from RS to TPM imaging. HT29s and T84s display a typical roundish shape, which is qualitatively maintained across microscopy modalities. Scale bars are 10 μm.

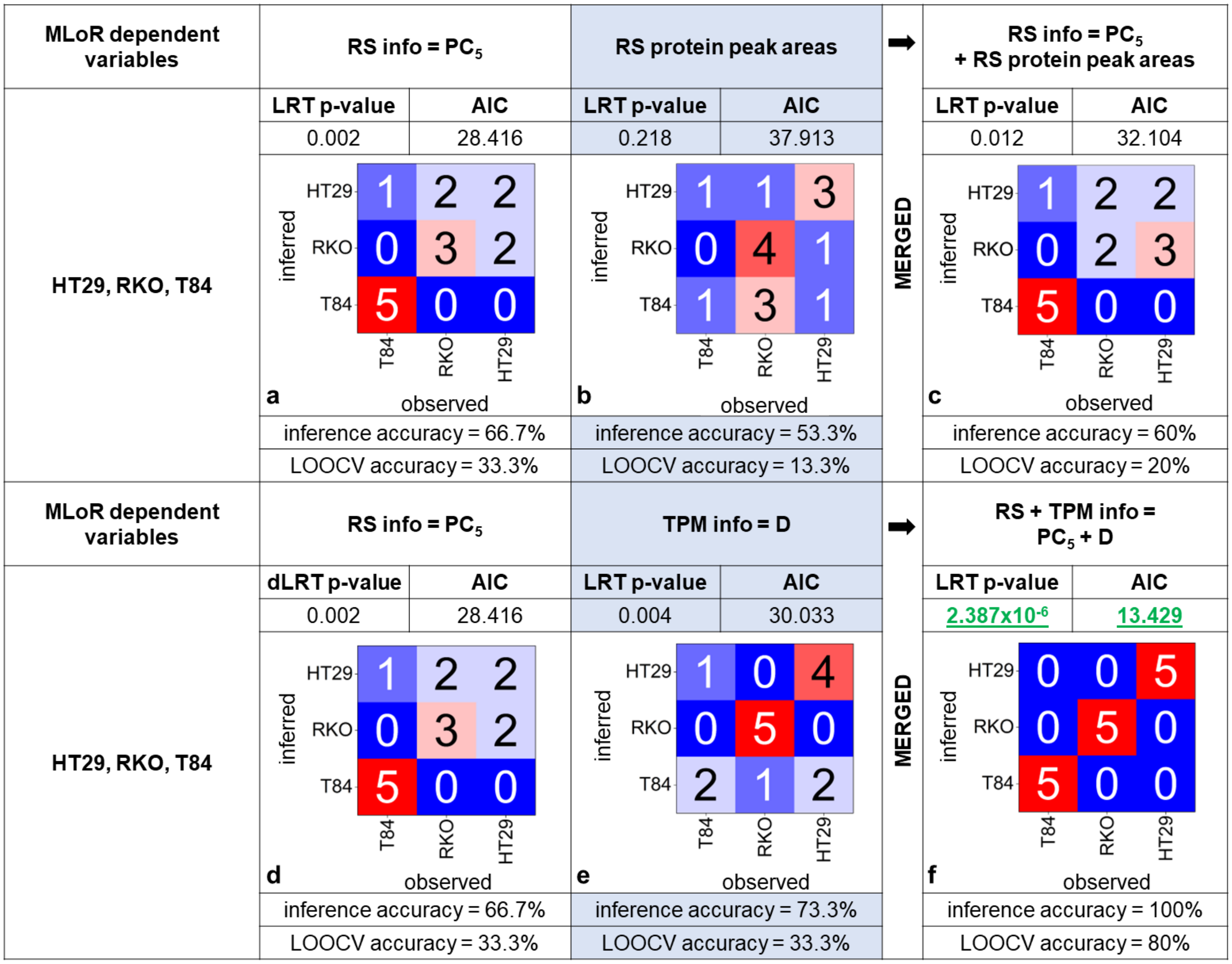

Supplementary Figure 8. Comparison of MLoR models performances using protein-related RS peaks instead of TPM-derived D to infer colon cancer types. Despite the positive correlation that RS peak areas show with TPM-derived cell DM, they cannot outperform the high phenotype inference accuracy obtained with the use of TPM-derived cell D in the reduced morpho-molecular model. In fact, protein-related RS peak areas, computed on cell-averaged Raman spectra, correlate significantly with the overall cell DM, not with the cell average D that is the one TPM-derived trait mostly distinguishing colon cancer cell types (Fig. 7f). The cell DM does not vary significantly among the very similar cancer cell types used through this work (Fig. 5b). Using an RS-based regressor that scales with the cells DM cannot benefit cell type inference, as quantified statistically in the figure. **a d** Reduced MLoR model

using only the most significant chemical information differentiating cell types, PC_5. **b** Reduced MLoR model using only protein-related RS peaks of phenylalanine at **Ω** = 1007 cm^{-1} and Amide I at **Ω** = 1660 cm^{-1} as independent variables, which were proved to have a significantly positive correlation (PCC = 0.977) with the cells DM (Fig. 8). **c** Enlarged RS-based MLoR model using all the information fed in A and B. This model uses only the chemical information, with the RS peak areas. **e** Reduced MLoR model using only the most significant morphological information differentiating cell types, D. **f** Best model in terms of LRT and AIC metrics, achieved coupling the one most significant chemical and morphological information, PC_5 and D. Despite the correlation between RS peak areas and the cells DM, related to the cells D via their V (*i.e.*, DM = D×V), the RS-based reduced information **c** cannot achieve equally high phenotype inference accuracy compared to the combined RS and TPM reduced model **f**.

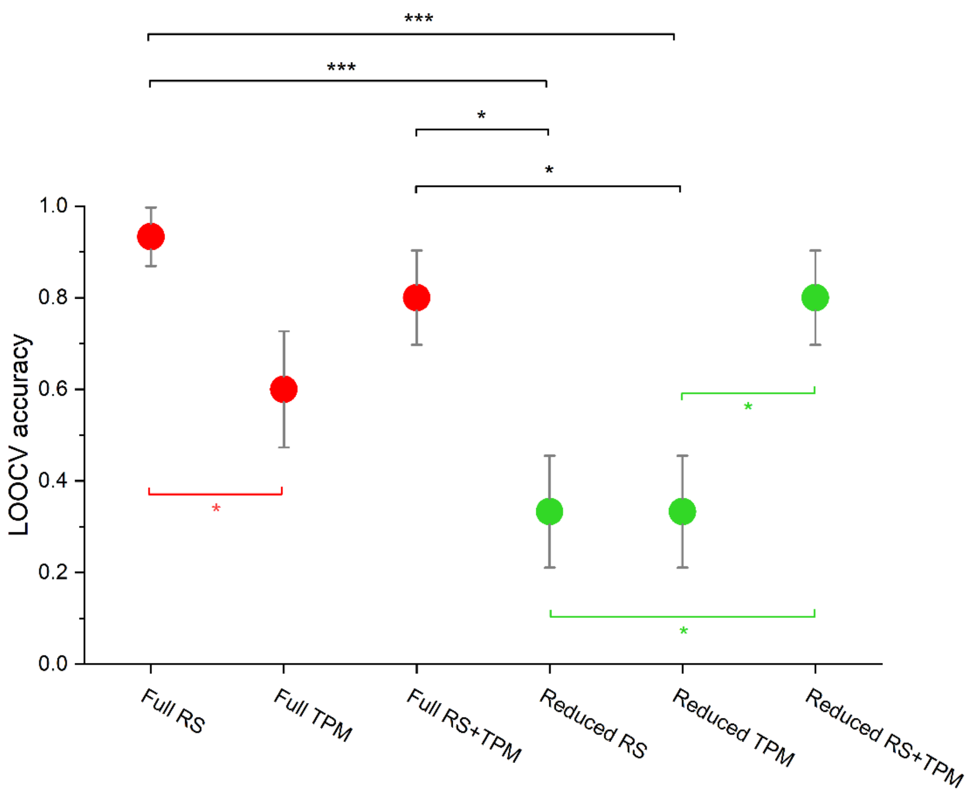

Supplementary Figure 9. The predictive power of the RS, TPM, and RS + TPM cell profiles, was evaluated through leave-one-out cross-validation (LOOCV) of MLoR models. LOOCV accuracy is displayed as mean ± standard error of the mean. For LOOCV, we trained the models introduced in Figure 7 as many times as the number of data points in our dataset, namely, 15. Each time, only one sample was used as a test set while the rest were used as the training set. Hence, the LOOCV accuracy was computed as the average accuracy of the 15 different models. This type of cross-validation ensures a good model predictive power evaluation in the case of a small dataset, as it will use more training samples in each iteration to enable learning better representations. LOOCV proves that the choice of a reduced RS + TPM dataset, comprising only the most significant RS (PC_5) and TPM (D) information, boosts cell phenotype prediction accuracy, making it as predictive as the one trained on the full set of significant RS (PC_2, PC_5, and PC_7), full significant TPM (D, Z, and Φ), and full significant RS + TPM (PC_2, PC_5, and PC_7, D, Z, and Φ) information. Indeed, no statistically significant difference in terms of LOOCV accuracy emerges

comparing the full RS, the full TPM, the full RS + TPM, and the reduced RS + TPM models. Notably, by using a reduced number of regressors, the advantage consists in maintaining high interpretability of the morpho-molecular traits driving cell type differentiation. Mainly, a drop in predictability is clear when considering the reduced RS and TPM information, with respect to all the other models. A two-sided Mann-Whitney U test was performed for statistical significance: * p-value ≤ 0.05; ** p-value ≤ 0.01; *** p-value ≤ 0.001. LOOCV accuracy of models trained with the full significant information for cell type discrimination is represented in red, and the one of models trained with a reduced subset of the most significant information is shown in green.

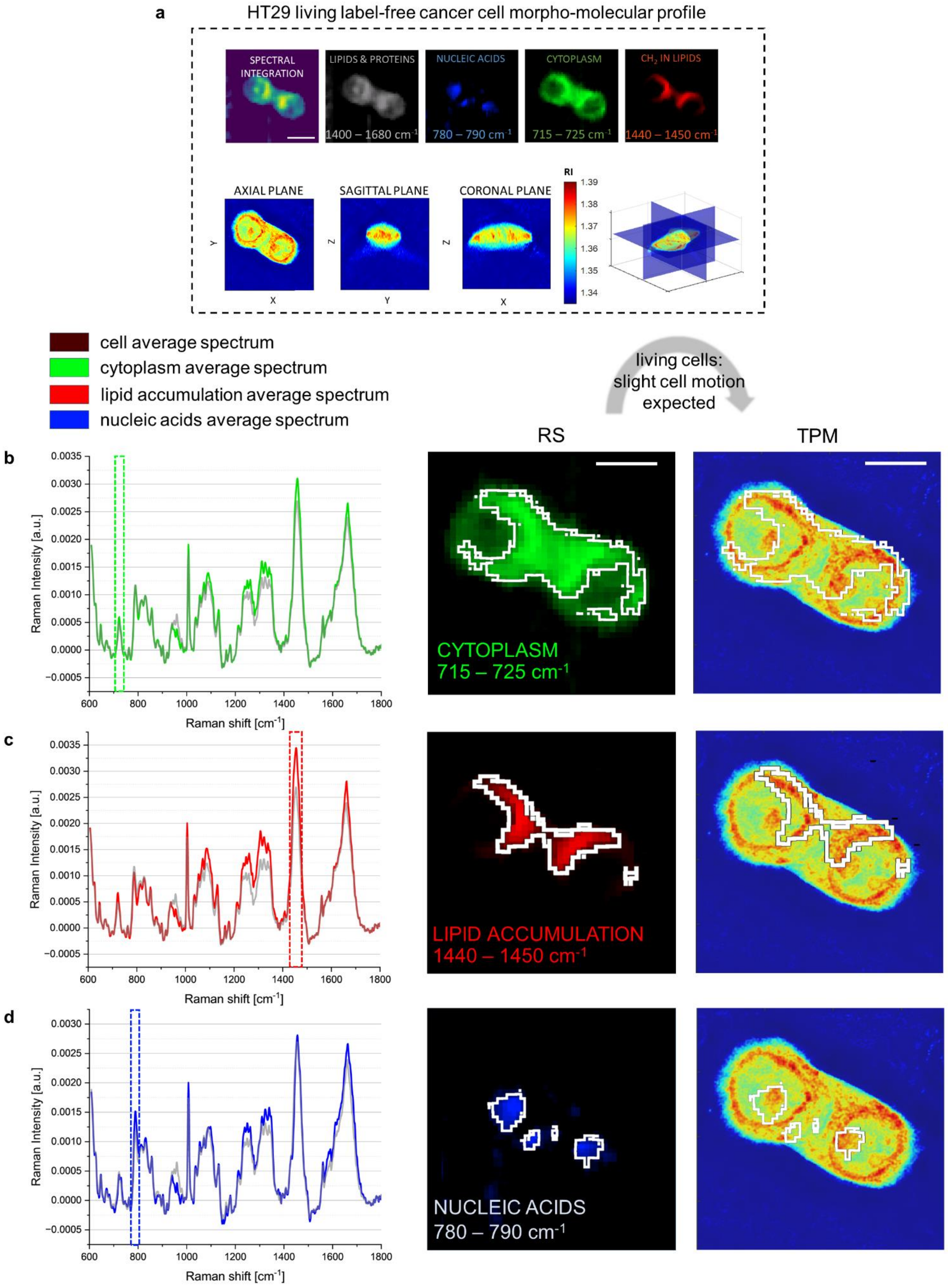

a
HT29 living label-free cancer cell morpho-molecular profile
SPECTRAL INTEGRATION
LIPIDS & PROTEINS
1400 – 1680 cm-1
NUCLEIC ACIDS
780 – 790 cm-1
CYTOPLASM
715 – 725 cm-1
CH2 IN LIPIDS
1440 – 1450 cm-1
AXIAL PLANE
SAGITTAL PLANE
CORONAL PLANE
RI
cell average spectrum
cytoplasm average spectrum
lipid accumulation average spectrum
nucleic acids average spectrum
living cells: slight cell motion expected
RS
TPM
b
c
d
Raman Intensity [a.u.]
Raman shift [cm-1]
CYTOPLASM
715 – 725 cm-1
LIPID ACCUMULATION
1440 – 1450 cm-1
NUCLEIC ACIDS
780 – 790 cm-1

Supplementary Figure 10. Co-registered RS and TPM allow for the chemical differentiation of intra-cellular organelles with similar refractive properties. Thanks to the co-registration of FOVs with equal dimension and comparable cell orientation, we prove that it is possible to segment subcellular structures based on the intensity of their RS fingerprint signal, then apply the same mask to retrieve their location in a central XY slice of the corresponding RI tomogram. With this, one can achieve the differentiation of intra-cellular structures revealed with high spatial resolution (lateral and axial resolutions of ≈110 nm and ≈356 nm, respectively) in TPM maps that feature the same RI. Notably, this intra-cellular investigation is not necessary for the sake of cell phenotyping, as we proved that the cell-averaged information, easier and quicker to be extracted, can effectively and significantly distinguish similar cell types in label-free and living conditions (Fig. 7). Also, due to an minor cell motion that we expect occurring over measurement time, as living cell conditions were ensured, an exact spatial overlap of RS and TPM maps is unlikely, whereas the cell-averaged morpho-molecular properties used for cell phenotyping through our study are robust to such slight movements. Scale bars are 10 μm. Signal thresholding and mask generation were carried out in Fiji-ImageJ. Signal threshold was automated through the IsoData function. **a** Morpho-molecular profile of a living and label-free human colon adenoma HT29 cancer cell in culture, likely undergoing cell division as suggested from its bi-nucleated structure. **b** Segmentation of cytoplasmic predominant portions of the target cell, through intensity thresholding of the Raman image at **Ω** = 715 – 725 cm^{-1}, corresponding to the Raman modes of $CN^+(CH_3)_3$ in lipids, choline group $N^+(CH_3)_3$ and phosphatidylcholine vibrations. The mask is applied to the central XY slice of the TPM map of the same cell, showing high spatial correspondence achieved despite minor cell motion. **c** Segmentation of the lipid accumulations in the cytoplasm of the HT29 cell, by intensity thresholding of the Raman hyperspectral map in the range **Ω** = 1440 – 1450 cm^{-1}, featuring the bending of CH_2 bonds in fatty acids. The lipid-related mask is applied to the central XY slice of the corresponding phase tomogram: despite the presence of similar RI structures throughout the cell, only a

subset of them can be identified as cytoplasmic lipid accumulations thanks to co-registered chemical mapping. **d** Segmentation of the nucleic acids-rich region of the cell via intensity thresholding of the Raman hypercube at $\Omega = 780 - 790$ cm^{-1}, featuring pyrimidines ring breathing, including both nucleobases of DNA and RNA cytosine and uracyl at $\Omega = 788$ cm^{-1}. The mask is applied to the central XY slice of the TPM map, showing evident spatial correspondence with the higher RI structures inside the cell nuclei, suggesting their identification with nucleoli.

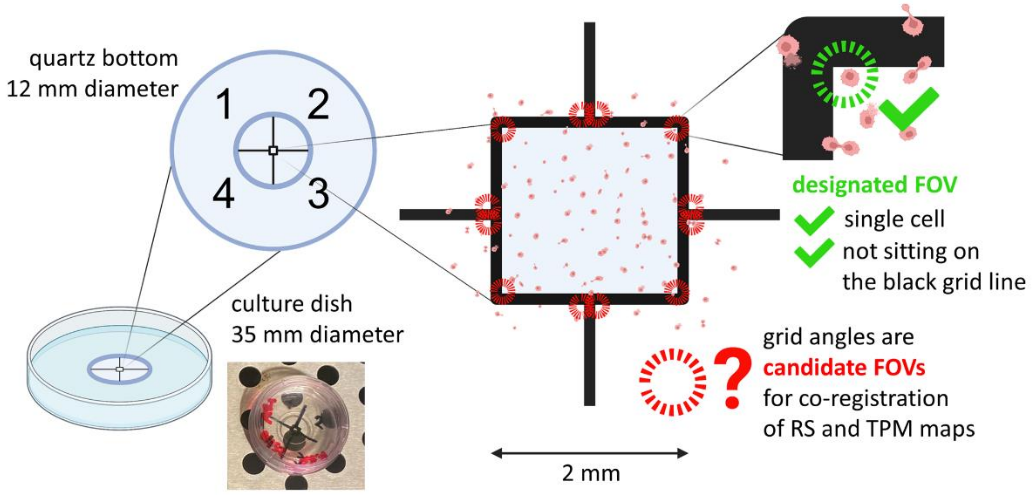

Supplementary Figure 11. Grid design on quartz bottom culture dishes for quick co-registration of single-cell FOVs between the RS and TPM systems. Ahead of cell culture, we carefully drew with the help of a ruler and a permanent ink pen the illustrated grid on the external side of quartz bottom disks of culture dishes, along with an indication of quadrant numbers. Then, once the dish was introduced into the incubation chamber of the RS system, we designated a target FOV among all the available grid angles (indicated with red dashed circles in the above sketch). The criteria for such a choice were: (i) having one single cell close to the grid angle, with a clear separation space between surrounding cells; (ii) all the cell parts were adhering onto a portion of the quartz substrate not featuring any black ink traces, which would induce light scattering and alter the collected signal. After RS acquisition, the dish was transferred into the incubation chamber of the adjacent TPM system. Here, the same grid angle was quickly identified with the help of quadrant numbers, and the previously imaged single cell could be localized next to the edges of the grid angle. With this, the target cell was quickly and easily identified, enabling co-registered TPM measurements. Due to the short transfer time from the RS system to the TPM system, we did not experience cell division starting and terminating through transfer time. From time to time, major cell movement or a neat cell division occurred through RS imaging, due to its longer acquisition time (*i.e.*, circa 1 hour) compared to systems switch or TPM imaging, which caused strong

blurring in the chemical map. For the sake of producing a clear morpho-molecular co-registration of RS and TPM images, to carry out a valid proof-of-concept study on the effectiveness of morpho-molecular phenotyping, such blurred RS images were excluded from the study. Conversely, minor cell reshaping and minimal cell movement were expected, as living cells were maintained in their physiological culture conditions inside incubation chambers. This behavior is totally acceptable and does not alter the soundness of the co-registration between the cell-averaged Raman spectrum and the cell-averaged TPM-derived traits. Notably, it supports the non-invasiveness of the methods. Such a minimal displacement can be clearly seen in Supplementary Figure 7 and Supplementary Figure 10.

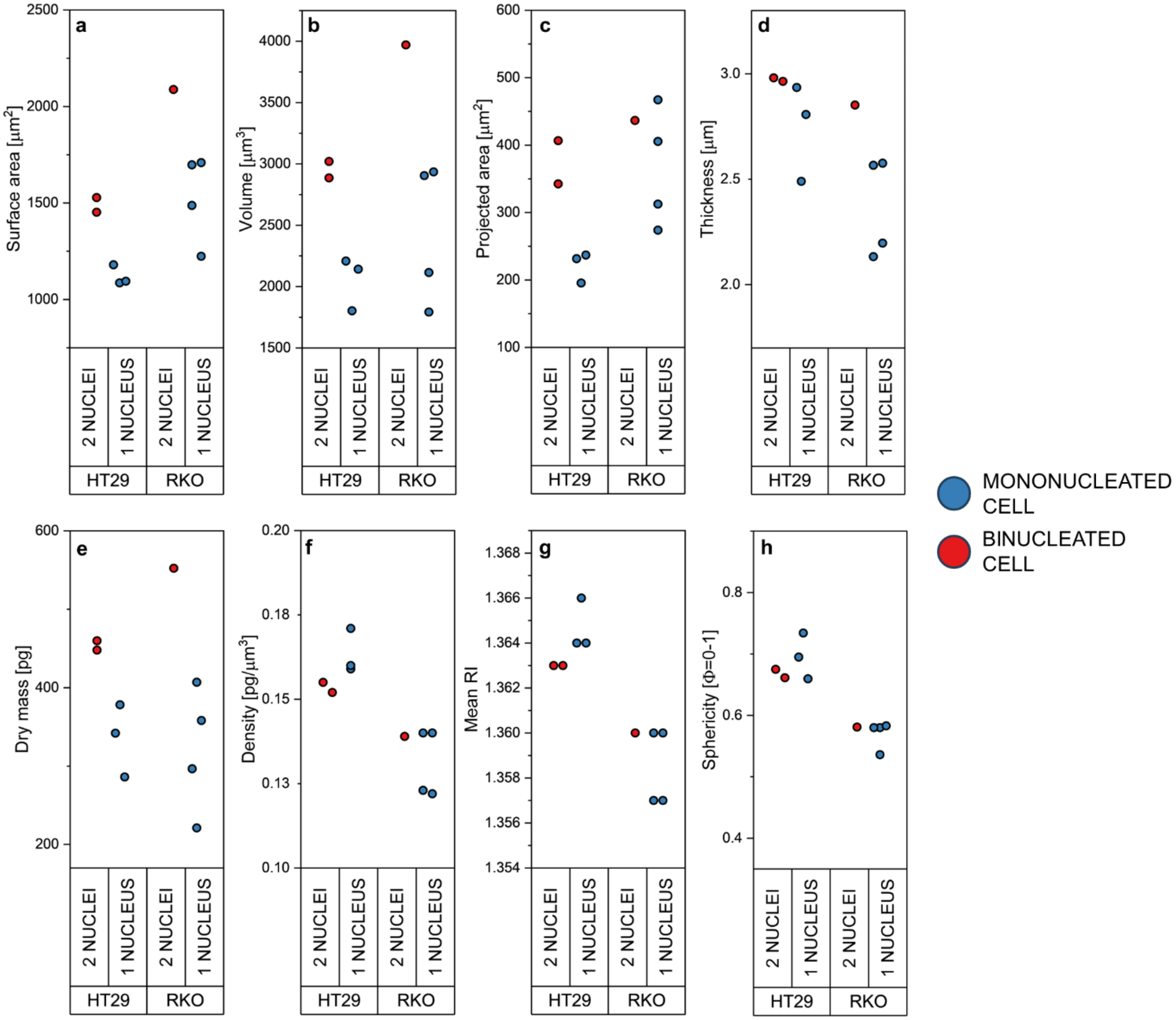

Supplementary Figure 12. Impact of different cell cycle phases on TPM observables. We did not synchronize cell cycles to make our methods agnostic to them and easily and directly applicable to pristine unmanipulated cells in culture. When cells are in a binucleated state, over their mitosis, the main phenotypic differences are given by an enlarged cell dimension (*i.e.*, **a** surface area (S) [μm^2], **b** volume (V) [μm^3], **c** projected area or footprint (A) [μm^2], and, in minor proportion, **d** cell thickness (T) [μm]), along with an increase in average cell dry mass (*i.e.*, **e** DM [pg]). **f g h** Other observables do not show appreciable variations among cell cycle phases. In fact, it is reasonable to observe enlarged cells through the mitotic phase, when two nuclei are generated in the cytoplasm and cells synthetize all the necessary

proteins to ensure a complete set of organelles for two daughter cells (namely, an increased value of total DM, which scales with the content of dry proteins present). We provide the data points of TPM traits of mono- and bi-nucleated colon cancer cells belonging to the same cell type, included in our dataset, from which such trends can be clearly evinced.

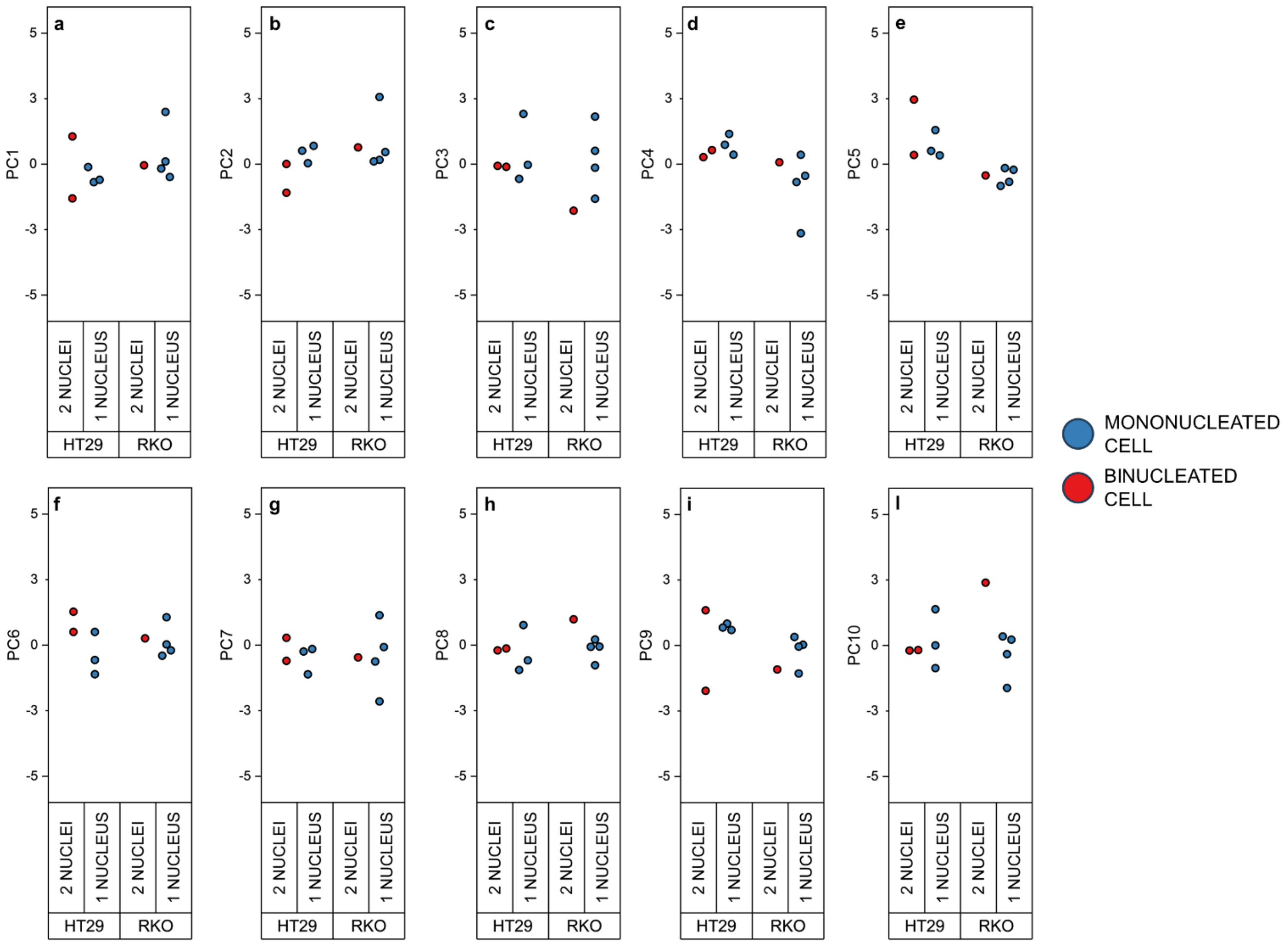

Supplementary Figure 13. Impact of different cell cycle phases on RS observables. We did not synchronize cell cycles to make our methods agnostic to them and easily and directly applicable to pristine unmanipulated cells in culture. While cells vary their TPM-derived dimensional and mass traits from interphase (generally mononucleated) to mitosis (insurgence of binucleated cells) (Supplementary Figure 12), RS fingerprints do not show signs of clear variations. Due to cells doubling their nucleus and synthetizing proteins to provide a complete set of cytoplasmic compounds and organelles to two daughter cells, the overall chemical composition of a doubling cell, as represented by the cell-averaged Raman spectrum, should be reasonably similar to the chemical composition of the mononucleated mother cell. **a - l** We provide data points of the first ten PCs of mononucleated versus binucleated cells, belonging to the same phenotype, as present in our dataset, representing 98.9 % of cumulative variance of the RS data

cloud. It is evident that the PC scores of binucleated cells do not clearly set apart from PC scores of mononucleated cells, in each PC considered: the overall chemical composition probed by RS is generally invariant to the sampled cell cycle phase. These results demonstrate the robustness of our method in being agnostic to cell cycles for the phenotyping task.
