# Supplementary material for: Label-free morpho-molecular phenotyping of living cancer cells by combined Raman spectroscopy and phase tomography
Source: Commun Biol. 2024 Jun 29;7:785. doi: 10.1038/s42003-024-06496-9 (PMC11217291; doi:10.1038/s42003-024-06496-9)
Supplement: Supplementary file 3 — Description of Additional Supplementary Files [file 42003_2024_6496_MOESM3_ESM.docx]

**Description of Additional Supplementary Files**

**File name**: Supplementary Data

**Description**: Datasets, analyses output data and graphs source data.
